# Decoding of movement direction using optical imaging of motor cortex

**DOI:** 10.1186/1471-2202-14-S1-P380

**Published:** 2013-07-08

**Authors:** Nicoladie D Tam, George Zouridakis

**Affiliations:** 1 Department of Biological Sciences, University of North Texas, Denton, TX 76203, USA; 2 Departments of Engineering Technology, Computer Science, and Electrical and Computer Engineering, University of Houston, Houston, TX, 77204, USA

## 

This study provides a computational scheme to decode intentional arm movement direction using optical imaging of the motor cortex for future implementation on a neuroprosthetic device that enables physically disabled patients to navigate a wheelchair using brain-derived signals. To this end, we developed a signal-processing algorithm for detecting movement direction from hemodynamic signals using functional near-infrared spectroscopy (fNIRS) recorded in human subjects during execution of a directional motor task. fNIRS has been shown to reflect hemodynamic responses in the cortex during execution of computational [1,4,6] and motor tasks [2], and can capture better regional cerebral blood volume (rCBV) changes [3] than blood-oxygen level dependent (BOLD) signals [5]. We used 64 spatially distributed optrodes to record from both hemispheres of motor cortices during free arm orthogonal movements in the *x *and *y *directions on a horizontal plane, extending the experimental findings reported earlier [7]. We then analyzed the spatiotemporal profiles of the 64-channel hemodynamic response to derive the direction of the movement executed from the motor cortex activation.

We employed four different measures of hemodynamic profiles -- oxy- (HbO_2_) and deoxy-hemoglobin (Hb), and their sum (HbO_2 _+ Hb) and difference (HbO_2 _- Hb) signals -- to correlate oxygen delivery, oxygen extraction, total blood volume delivered, and total oxygenation with a series of specific movements to identify the direction of the intentional movements. This analysis provided a unique representation of the different hemodynamic components of the localized neuronal populations in the motor cortices underlying the optrodes.

Our results show that the four measures of hemodynamic response may be coupled in one movement direction and decoupled in another, for the same subject. That is, oxygen delivery, extraction, total blood volume, and oxygenation do not necessarily co-vary (increase or decrease simultaneously) for all movement directions, but for some directions, they may be decoupled; e.g., oxygen delivery may increase while, at the same time, oxygen extraction may decrease. This suggested that oxygen extraction may outpace oxygen delivery due to high oxygen demand of the underlying neural tissues, resulting in decoupling of the oxygen delivery and extraction variables. Thus, if decoding of movement direction relies on a single hemodynamic measure, the latter is not sufficient to identify the movement direction uniquely. Instead, a combination of all four measures of hemodynamic signals is needed, and it can be extracted from the temporal profiles of neural activation and deactivation that represent temporal coupling and decoupling of oxygen delivery and extraction. Overall, our experiments demonstrate the feasibility of decoding intentional movement direction using computational analysis of optical recordings from the motor cortex, instead of implanting microelectrodes, and support a future implementation of a hands-free neuroprosthetic device for the physically disabled to navigate a wheelchair unassisted.
